# Shoot-root signal circuit: Phytoremediation of heavy metal contaminated soil

**DOI:** 10.3389/fpls.2023.1139744

**Published:** 2023-02-20

**Authors:** Shiyan Bai, Xiao Han, Dan Feng

**Affiliations:** ^1^ College of Biological Science and Engineering, Fuzhou University, Fujian, China; ^2^ Biotechnology Research Institute, Chinese Academy of Agricultural Sciences, Beijing, China

**Keywords:** root, shoot, phytoremediation, heavy metal, soil

## Abstract

High concentrations of heavy metals in the environment will cause serious harm to ecosystems and human health. It is urgent to develop effective methods to control soil heavy metal pollution. Phytoremediation has advantages and potential for soil heavy metal pollution control. However, the current hyperaccumulators have the disadvantages of poor environmental adaptability, single enrichment species and small biomass. Based on the concept of modularity, synthetic biology makes it possible to design a wide range of organisms. In this paper, a comprehensive strategy of “microbial biosensor detection - phytoremediation - heavy metal recovery” for soil heavy metal pollution control was proposed, and the required steps were modified by using synthetic biology methods. This paper summarizes the new experimental methods that promote the discovery of synthetic biological elements and the construction of circuits, and combs the methods of producing transgenic plants to facilitate the transformation of constructed synthetic biological vectors. Finally, the problems that should be paid more attention to in the remediation of soil heavy metal pollution based on synthetic biology were discussed.

## Introduction

The global soil heavy metal (HM) pollution is increasing (Outridge et al., 2018). Heavy metals in the environment enter organisms along the food chain, causing harm to organisms and human bodies (Moynihan et al., 2017). For example, “Minamata disease” in Japan is caused by Hg pollution (Eto, 2000). Both physical remediation and chemical remediation have the disadvantages of high treatment cost, large treatment project (Ferrucci et al., 2017; Mu'azu et al., 2018; Asadollahfardi et al., 2021), and disturbing the soil microenvironment (Zhou et al., 2015). Although microbial remediation can effectively deal with heavy metal pollution in soil, it also has some shortcomings such as demanding environmental conditions (such as specific pH, temperature, etc.) (Cabral et al., 2013). Nanomaterials have excellent adsorption properties for heavy metals (Marques Neto et al., 2019), but the interaction mechanism between them remains to be studied (Hizhnyi et al., 2017; Zhang et al., 2021), so there are limitations when they are used to remove heavy metals. It is urgent to find appropriate methods to control soil heavy metal pollution.

Phytoremediation has low cost and simple operation, which is suitable for dealing with heavy metal pollution in large areas of soil. It is expected to reduce the content of heavy metals in contaminated soil to a safe level for a long time, so as to eradicate the problem of heavy metal pollution in soil (Pilon-Smits, 2005). Hyperaccumulators are commonly used in phytoremediation (Li et al., 2018). However, the discovered hyperaccumulators have poor environmental adaptability and enrichment specificity and are lack of critical biomass for effective phytoremediation (Mijovilovich et al., 2009). Synthetic biological methods to improve plant tolerance and toxic metal accumulation have great potential in phytoremediation.

The behavior of these pathways in plants can be predicted, regulated and finally programmed (Schwille, 2011). Unlike traditional method, synthetic biology is a new way to build modules with new functions (Liu and Stewart, 2015). In this paper, the comprehensive process of soil heavy metal pollution control and the strategies of synthetic biology involved are proposed, and the latest progress of experimental technologies that contribute to synthetic biology and plant gene transformation is summarized. This review also discusses the potential challenges of applying synthetic biology to phytoremediation. Efforts should be made to formulate breeding plans to improve the characteristics of natural hyperaccumulators, and cultivate these characteristics into non-food, high accumulation, high biomass plants for phytoremediation of heavy metals.

## Synthetic biology devotes to intelligent improvement of ideal traits

Synthetic biology combines biotechnology and engineering ideas to connect genes into a network, enabling cells to complete various tasks of artificial design (Khalil and Collins, 2010; Cameron et al., 2014). Compared with bacteria, yeast and mammalian cells, plant synthetic biology is still in its infancy (Liu and Stewart, 2015). Moreover, phytoremediation of heavy metal contaminated soil also has some problems, such as mixed heavy metal pollution. *Thlaspi caerulescens*, a cadmium/zinc hyperaccumulation plant, is sensitive to Cu toxicity, which is a problem in the application of this plant to the remediation of cadmium/zinc contaminated soil with Cu (Mijovilovich et al., 2009).

**Figure 1:**
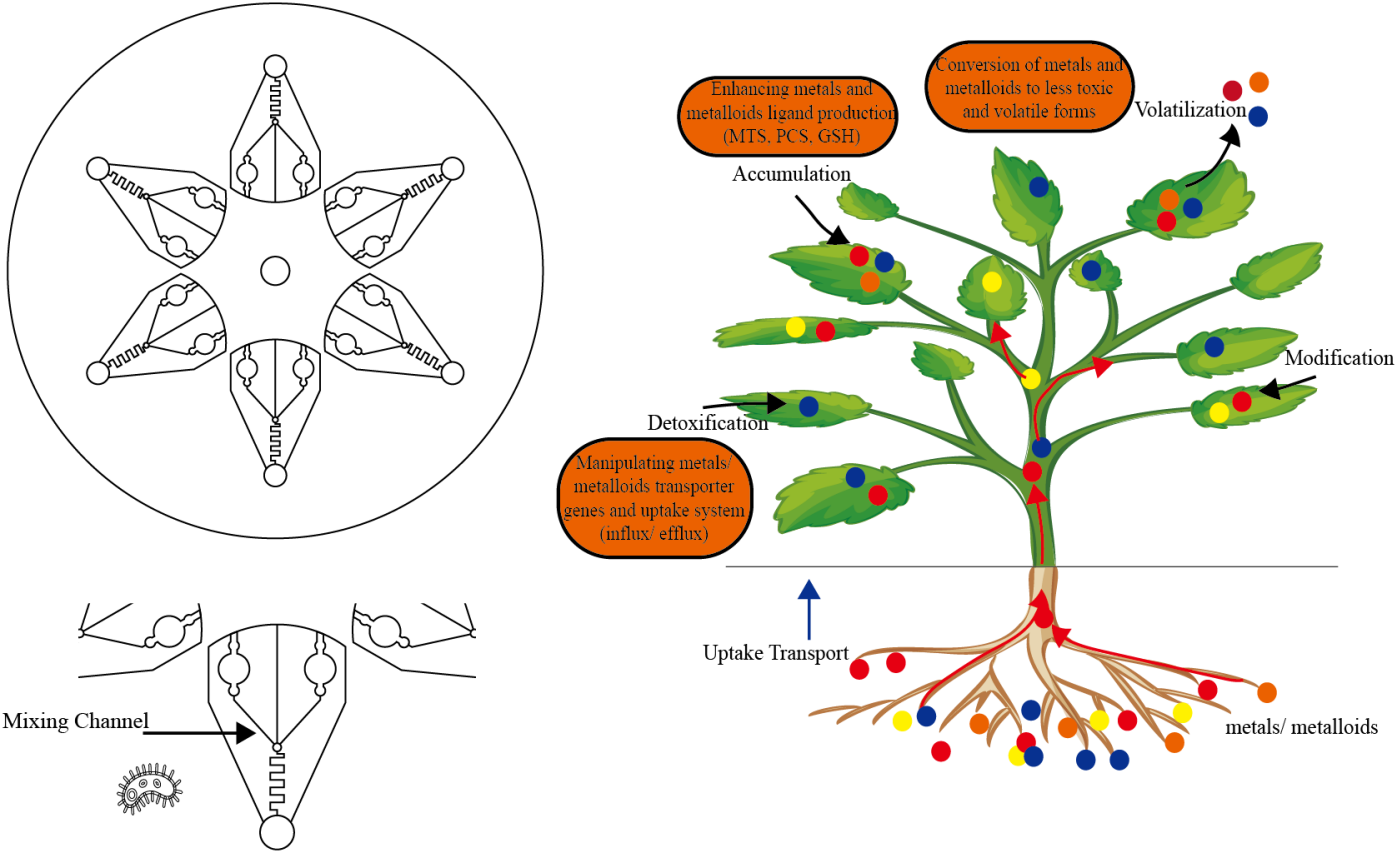
Strategies for phytoremediation of heavy metal contaminated soil.

Although plant sensors have been developed to use chlorophyll destruction to achieve visualization (Antunes et al., 2011), due to the limitations of plants, such as their bright colors and spontaneous fluorescence, and in view of the large variety of heavy metals, we propose to implement the strategy of “microbial biosensor detection-phytoremediation-heavy metal recovery” for soil heavy metal pollution (**Figure 1**). Synthetic biological elements are constantly added to the gene circuit of microbial biosensors and phytoremediation, so that their functions are more and more abundant and their performance is constantly improved.

## Heavy metal microbial biosensor

**Figure 2:**
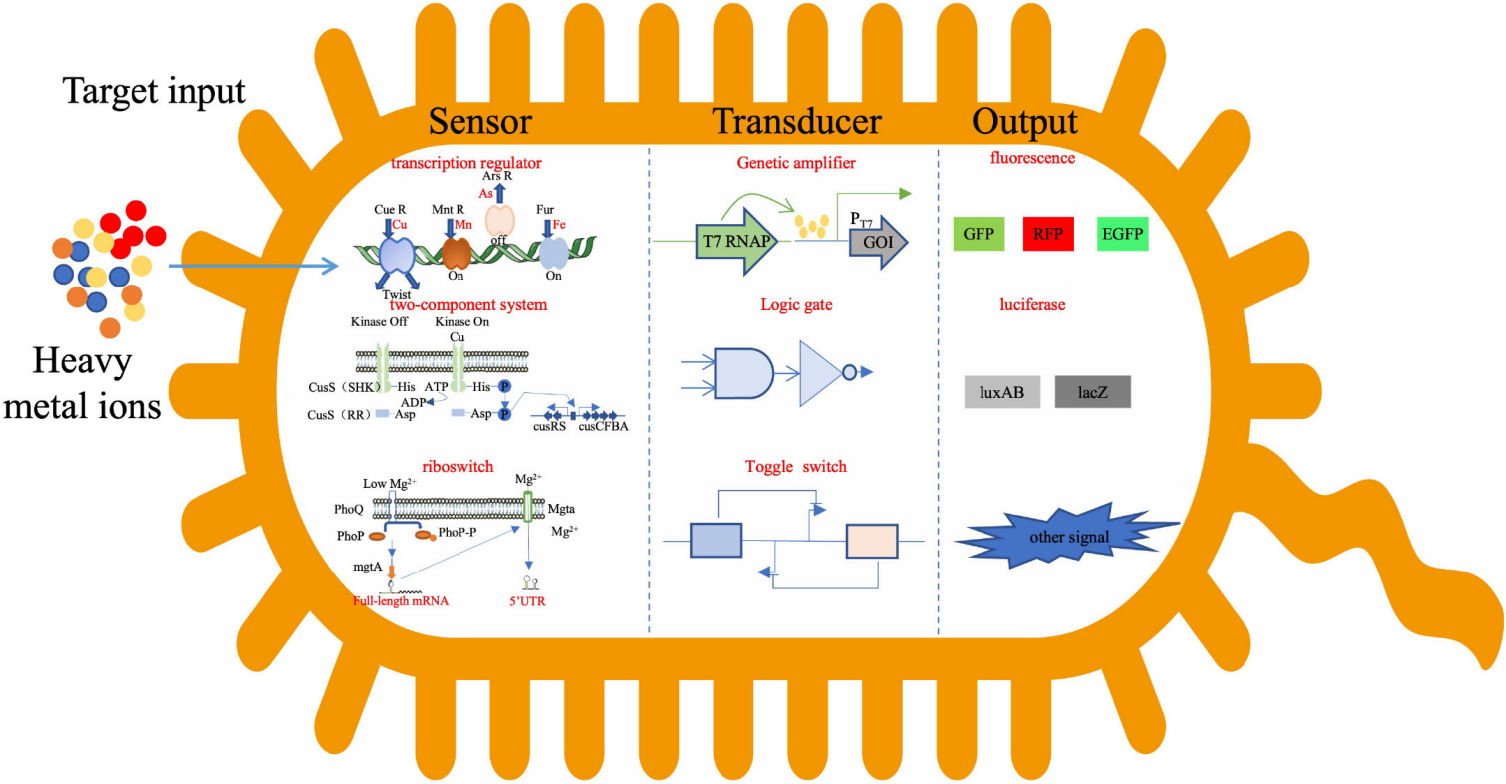
Signal transduction in cell.

Whole cell microbial biosensor refers to using microbial cells as sensing elements to convert the collected molecular information to light, electricity and other signals. The signal intensity is proportional to the content of the substance to be measured (Du et al., 2019), so as to achieve quantitative and qualitative dynamic monitoring of the substance to be measured. The diversification of detection targets is one of the development trends of microbial biosensors. In order to achieve functional integration of multi target detection, more gene elements must be installed in the gene loop of microbial biosensor, which leads to increasingly complex gene loop. Synthetic biology provides theoretical and technical support for the integration and optimization of gene circuits to ultimately achieve programmed/customized sensitivity, specificity, and dynamic ranges of sensors to meet their real world detection requirement (**Figure 2**).

The first reported gene engineering cell biosensor was responsive to aromatic hydrocarbon contamination (King et al., 1990). After that, many heavy metal microbial biosensors were developed. AND, OR, NOR, NAND, XOR and other complex modular logic gate structures are designed and constructed, and used for the development of multi-level gene circuit type whole cell microbial biosensors (Wang et al., 2013; Saltepe et al., 2018). The whole-cell-based biosensor consists of sensing elements and reporting elements, all of which are located in the chassis cell to realize the designed gene circuit (Harms et al., 2006).

The microbial biosensor uses autologous cells as sensing units to sense the measured objects and convert them into recognizable signals according to certain rules (Yagi, 2007). Due to the easy culture, rapid reproduction and relatively simple metabolism of microorganisms, microbial biosensors have natural advantages. However, the microorganisms commonly used to construct microbial biosensors are limited to bacteria, yeast, cyanobacteria, green algae and other microorganisms (Ma et al., 2022).

The sensing elements of heavy metal microbial biosensor mainly include heavy metal responsive transcription factor/transcription regulator (Brown et al., 2003; Busenlehner et al., 2003; Fang and Zhang, 2022), two-component system (Leonhartsberger et al., 2001) and riboswitch (Cromie et al., 2006). The regulatory structure of transcription factors/transcription regulators is the most widely studied and applied gene circuit sensing element at present. These proteins have two functional domains, namely ligand-binding domain (LBD) and DNA-binding domain (DBD). The LBD is the signal receiving module of the sensing element, which determines the specificity and diversity of the ligand. The DBD is a signal conversion module, which can specifically identify transcription factor/transcription regulator binding sites. Metal responsive transcription regulators (i.e. metalloregulators) have different families: ArsR/SmtB, MerR, CsoR/RcnR, CopY, DtxR, Fur, NikR, etc (Pennella and Giedroc, 2005; Osman and Cavet, 2010). At present, MerR family proteins and ArsR/SmtB family proteins are mainly used in the construction of microbial biosensors. Transcription factors/transcriptional regulators have clear functional domains, which can be separated and recombined in a modular way to a certain extent. Based on the modular structure of natural transcription factors/transcriptional regulators, artificial transcription factors/transcriptional regulators (ATF/ATR) can be designed and constructed. ATF/ATR integrates different LBDs and DBDs and directly targets key gene regulatory networks that govern intended downstream application (Tungtur et al., 2007). Promoters containing transcription factor binding sites are also the focus of research. The response performance can be adjusted by changing the strength of promoters (Xu et al., 2020), the location (Dabirian et al., 2019)and number (David et al., 2016) of transcription factor binding sites.

The report element is a report gene that can be monitored. At first, luciferase was used to construct the microbial biosensor. Later, fluorescent protein became the mainstream reporting element, and the programmable design of heavy metal microbial biosensor was realized. A series of constitutive promoters are used to regulate the expression level of MerR family proteins, which are used as regulators in the genetic circuit to regulate the detection sensitivity to control the expression of fluorescent protein eGFP. Based on this, a heavy metal microbial biosensor with adjustable sensitivity is designed (Guo et al., 2019). The concentration of heavy metal ions in the environment can be converted by measuring the fluorescence intensity of microbial biosensor cells (Du et al., 2019). However, as far as the sensitivity to heavy metals is concerned, the performance of luciferase is better than that of fluorescence (Huang et al., 2015). Pigment genes are also used to construct microbial biosensors (Fujimoto et al., 2006).

Integrated with micro/nano technology, some systems have been developed to optimize microbial biosensors and make them easy to use (Rothert et al., 2005; Buffi et al., 2011; Kim et al., 2015). With the discovery of new mechanisms of recognition and allosteric of metalloregulators (Liu et al., 2019; Fang et al., 2021), microbial biosensors are easy to use and have higher sensitivity and faster detection speed.

## Phytoremediation

### Hyperaccumulator

Hyperaccumulator is the basis of phytoremediation. Hyperaccumulator can grow normally in the soil with high concentration of heavy metals, and accumulate heavy metals in the aboveground parts with a concentration hundreds or even thousands of times as high as that of ordinary plants (Sytar et al., 2021).

In 2015, the online Global Hyperaccumulator Database (www.hyperaccumulators.org) was established (Reeves et al., 2018; Sytar et al., 2021). The word heavy metal hyperaccumulator first appeared in 1976, when Sebertia acuminata was discovered, which can absorb and enrich nickel (Ni) in soil (Jaffré et al., 1976). Later, many hyperaccumulator were found, such as Arabidopsis halleri (Briskine et al., 2017), Alfalfa (Wang et al., 2021), and Sedum alfredii (Qiong et al., 2021). Pteris. Vittata L is the first reported hyperaccumulator of As in the world, and also the first reported fern with super enrichment function. Unfortunately, the website does not provide services at present.

It is necessary to continue and accelerate the discovery of heavy metal hyperaccumulator. As heavy metal hyperaccumulators only exist or mainly exist on metal bearing soil, they are threatened by habitat loss with the reduction of mineral resources (Lange et al., 2017). Therefore, it is necessary to continue to identify hyperaccumulator species and other metal tolerant plants in order to study and utilize their unique physiological mechanisms and provide a basis for the practical application of phytoremediation technology. However, we advocate that native plant species should be used as much as possible to avoid the possible spread of invasive species.

Some crops can enrich heavy metals. As and Pb exceeding the standard were detected in the harvestable part of corn growing near the tailings (Armienta et al., 2020). *Brassica juncea* L. showed the ability to enrich copper, cadmium and lead from soil (Goswami and Das, 2015; Gonzaga et al., 2018; Gurajala et al., 2019). The edible part of rice contains a lot of arsenic (Meharg, 2004; Dolphen and Thiravetyan, 2019). When wheat and some vegetables are planted in arsenic contaminated farmland, a large amount of arsenic is accumulated in their edible parts (Saeed et al., 2021; Shukla et al., 2022). However, we emphasize that the synthetic biological chassis carrier for phytoremediation should not use existing crops to ensure that the products produced do not enter the food chain.

## Application of synthetic biology to the improvement of existing hyperaccumulators

At present, more than 700 species of hyperaccumulators have been found, but the popularization of phytoremediation technology in practice is still restricted by many aspects. Each plant often accumulates only one or a few heavy metals, and shows some poisoning symptoms for other heavy metals with high concentrations in the soil. There are great limitations in the treatment of soil contaminated by multiple heavy metals (Wilson-Corral et al., 2012). The poor enrichment ability of heavy metals in soil, slow growth rate and small dry matter weight per plant bring great difficulties to the practical application of production (Bian et al., 2020).

The response of hyperaccumulators to heavy metals is closely related to a variety of genes, whose expression products mainly include metal transporter (Pence et al., 2000), phytochelatin synthase (PCS), metallothioneins (MTs) and metal reductase (Ellis et al., 2006). These proteins play an important role in the absorption, transport and partition of heavy metals in plants (Milner et al., 2013). The high level expression of TgMTP1 gene in the heavy metal hyperaccumulator Thlaspi goesingense is the reason for its strong ability to accumulate metal ions in vacuoles (Persans et al., 2001).

Genetic engineering tools have been successfully used to develop transgenic hyperaccumulators. By overexpressing metallothionein, phytochelatin, metal transporter and antioxidant enzymes in plants, it has been successfully demonstrated that the ability of phytoremediation is improved (Zanella et al., 2016; Balzano et al., 2020). Transferring genes responsible for hyperaccumulative phenotypes to plants with higher aboveground biomass is considered to be a feasible potential way to enhance phytoremediation (Brown et al., 1995; Rugh et al., 1998). However, the response of plants to heavy metals involves various proteins, amino acids, citric acid (Lu et al., 2013), etc. These substances are interrelated and interact to form a complex and huge signal network to regulate the whole growth and development process of plants. Only operating on individual genes can not create ideal hyperaccumulators, and even cause plants to be highly sensitive to heavy metals (Wojas et al., 2008). In addition, a single element can only enable transgenic organisms to obtain one of the functions of absorption, transport and transformation, while the synergy of multiple gene elements can obtain more potential repair species.

The most important core idea of synthetic biology is standardization. Different genes are designed into modular engineering elements. Through the design and assembly of the elements, a circuit that functions with time and space can be obtained (Endy, 2005). Synthetic biology of higher plants is emerging. The synthetic biology resource library iGEM (https://parts.igem.org/MainPage), SynbioML@TJU (http://www.synbioml.org/), Registry and database of bioparts for synthetic biology (https://www.biosino.org/rdbsb/), etc. constantly updated and supplemented the tested plant elements. SBOL has been updated to version 3.0 (Baig et al., 2020). Plant MoClo Syntax, a plant cloning system that can easily assemble complex vectors, has been established (Engler et al., 2014; Hussey et al., 2019).

Complex synthetic gene circuits have been achieved in plants. Recently, the first stable reprogramming synthetic gene circuit in plant cells has been realized. In this system, a series of key gene circuit functions were first established. Then, using recombinase and plant control elements, a series of operational logic gates were developed. The YES, OR and gates were used to activate transgenes, and the NOT, NOR and NAND gates were used to inhibit transgenes; A NIMPLY B gate combining activation and suppression is also realized. Through the use of gene recombination, these circuits have produced stable long-term changes in the expression and recording of past stimuli, proving the practicability of programmable manipulation of transcriptional activity in complex multicellular organisms (Lloyd et al., 2022). Gene circuits used to change root structure predictably have also been developed, which based on a series of synthetic transcriptional regulators developed for plants (Brophy et al., 2022). The application and development of hyperaccumulator in Phytoremediation will be promoted by using synthetic biological techniques to design and develop ideal hyperaccumulator with strong enrichment capacity.

## Heavy metal recovery in phytoremediation

Phytoremediation uses phytoextraction. If the plants that have absorbed heavy metals are not properly treated, the problem of environmental pollution still exists (Zhong et al., 2015). Research shows that plants are “bio factories” of metal nanoparticles. *Brassica juncea* can reduce silver ions and gold ions to form silver nanoparticles and gold nanoparticles with a particle size of 2~100 nm, and the output of the nanoparticles is affected by the amount of reducing sugar (Beattie and Haverkamp, 2011). After soybean (*Glycine max*) and rice (*Oryza sativa* L.) were exposed to silver ions, silver nanoparticles were also detected in plants, indicating that they were formed *in vivo (* Li et al., 2017). A variety of metal nanomaterials can be synthesized by genetically engineered microorganisms (Kang et al., 2008; Choi et al., 2018). MTs and PCS are commonly used in these modifications (Kang et al., 2008; Choi et al., 2018). Therefore, based on the development of sequencing technology and the application of synthetic biology in phytoremediation, it is possible to build a circuit in plants to generate metal nanoparticles at room temperature and pressure to achieve the classified recovery of heavy metals after adsorption.

## New technologies for developing synthetic biology modules

Synthetic biology technology may strongly support the development of phytoremediation. There are a lot of undeveloped element resources in plants. At present, most of the regulatory elements verified by experiments and characterized by functions come from rice, Arabidopsis and other model plants. There are still a large number of regulatory elements in non model plants waiting for further exploration and development.

In the past decades, although some progresses have been made in studying the mechanism involved in the interaction between organisms and heavy metals using molecular biological techniques (Hu et al., 2005; He et al., 2011; Hossain and Komatsu, 2012; Muralidharan et al., 2012), the potential of phytoremediation has still not been fully exploited. Many emerging biotechnologies help to identify components and redesign circuits.

### Third-generation sequencing

The third generation sequencing technology (TGS), also known as single molecule sequencing, has the advantages of long reading, single molecule and real-time sequencing (Schadt et al., 2010). At present, the mainstream platforms are single molecule real-time sequencing (SMRT-seq) and nanopore sequencing. Nanopore sequencing has been applied to DNA (Clarke et al., 2009; Manrao et al., 2012) and RNA (Wanunu et al., 2010) sequencing at the single molecular level, and has rapidly become the preferred technology for new genome assembly and structural variation identification (Schmidt et al., 2017; Fuselli et al., 2018; Tan et al., 2018). It can greatly improve the quality and integrity of sequencing data (Ding et al., 2020) and identify splice isoforms (Byrne et al., 2017; Depledge et al., 2019). The third generation of long reading sequencing enables us to obtain epigenome/epigenetic transcriptome data with single nucleotide resolution, which can be used to directly detect DNA and RNA modifications (Zhu et al., 2018; Tellgren-Roth and Couturier, 2022), and has become a common method to identify epigenetic modifications in plants (van Dijk et al., 2018; Zhao et al., 2020). This technology can accelerate the discovery of functional genes and can be used to manufacture biological components used in synthetic biology.

### Single-cell omics

In the past ten years, the methods of single-cell omics have completely changed our understanding of the cell and molecular composition of life systems. The sequence and structure analysis of genome (Luo et al., 2019), transcriptome (Tang et al., 2009), epigenetic modification (Zhu et al., 2019; Luo et al., 2020), chromatin accessibility (Marand et al., 2021) and 3D genome structural characteristics (Zhou et al., 2019; Ulianov and Razin, 2022) under single cell resolution are helpful to explain biological development laws and physiological mechanisms. Smart-seq2 is one of the most widely used single cell full-length transcriptome sequencing technologies (Picelli et al., 2014). Combined with Nanopore long-read sequencing, scRNA-seq has been improved to flsnRNA-seq, which can analyze large-scale full-length RNA at a single-nucleus in a protoplasting-free manner (Long et al., 2021). Based on TGS platform, scNanoATAC-seq technology was developed, which is a long-read single-cell ATAC sequencing method on Nanopore sequencing platform for simultaneously detecting chromatin accessibility and genetic variation in a single cell (Hu et al., 2022). Live-seq technology enables a single cell to maintain cell viability after transcriptome sequencing, which is the first time to achieve continuous observation of the whole gene expression in living cells (Chen et al., 2022). Single cell multiomics sequencing directly relates different omics information at the same time, and further studies single cell status and molecular regulation mechanism (Guo et al., 2017; Lee et al., 2020; Thibivilliers and Libault, 2021).

Spatial transcriptomics uses *in situ* capture technology (Ståhl et al., 2016), which can reveal the spatial distribution of various cell types in tissues, the interaction between various cell populations, and map gene expression in different tissue regions (Chen et al., 2021; Liao et al., 2021). Stereo-seq is the technology with the highest spatial resolution at present (Chen et al., 2022). DBiT-seq realizes the joint measurement of spatially distributed mRNAs and proteins (Liu et al., 2020). Spatial profiling of chromatin accessibility (spatial-ATAC-seq) (Deng et al., 2022b) and histone modifications (Spatial-CUT&Tag) (Deng et al., 2022a) provides new opportunities for understanding life activities. In a word, the methods of single-cell omics enable people to accurately analyze various genetic variations and provides a basis for designing and manipulating various mechanisms. However, the application of these technologies in plants needs further exploration.

### Genome editing technology

CRISPR–Cas-mediated genome editing efficiently and accurately simplifies, inserts or reconstructs the synthetic circuits and the genome of chassis organisms (Esvelt and Wang, 2013), providing strong support for the development of synthetic biology. It has been widely studied in many plants (Jiang et al., 2013; Li et al., 2013; Hu et al., 2017; Rodríguez-Leal et al., 2017). In addition, it has been able to replace large fragments of more than 100 kb (Wang et al., 2016) and knock out and knock in multiple genes at the same time at multiple targets (Jiang et al., 2016).

Based on dCas9 (Cas9 with H840A and D10A mutations) and different transcriptional regulatory domains, CRISPRi (CRISPR interference) and CRISPRa (CRISPR activation) achieve the goal of gene expression regulation without changing the target sequence (Maeder et al., 2013; Qi et al., 2013). dCas9 has also been developed as a tool for regulating gene expression at the level of epigenetic modification (Hilton et al., 2015). Based on the SunTag-dCas9-TET1cd system of Arabidopsis thaliana, an epigenetic editing system targeting the removal of rice genomic DNA methylation was constructed, which successfully reduced the DNA 5mC level of OsFIE1 gene and caused dwarfing phenotype (Tang et al., 2022).

Base editing technology allows the direct and stable conversion of target DNA or RNA bases into substitutes in a programmable manner, without the need for nucleic acid strand breaks and donor templates. The emergence of CBE (C•G to T•A base pair conversion) marks the birth of this technology, and it is found that the efficiency of nCas9(Cas9 with D10A mutation) is higher than that of dCas9, which is currently commonly used (Komor et al., 2016). ABE (adenine base editor) mediates the transformation from A•T to G•C in genomic DNA (Gaudelli et al., 2017). There are numerous SNP (Single Nucleotide Polymorphism) in plants, which are closely related to plant disease resistance and growth (Henry and Edwards, 2009; Malmberg et al., 2019). Plant base editing tools have been developed, and different CBEs may have different editing efficiency for the same region of the genome. Multiple CBE-ABE plant double base editors can edit different bases at the same time (Xiong et al., 2022). In plant epigenetics, APOBEC3BCtd-nCas9, a single base editor with high efficiency for editing methylcytosine, has been obtained (Liu et al., 2022).

At present, Prime Editor (PE) has realized free conversion of all 12 single bases and precise insertion/deletion of specific base sequences without relying on DNA templates (Anzalone et al., 2019). This method has been applied to some plants (Butt et al., 2020; Hua et al., 2020; Li et al., 2020; Lu et al., 2021), and has obtained tools with higher prime-editing efficiency (Jiang et al., 2020; Lin et al., 2021). The establishment of STEME (saturated targeted endogenous mutagenesis editor) has realized the directional evolution of OsACC gene in rice, thus obtaining herbicide resistance mutation, which provides the possibility for rapid acquisition of beneficial agronomic traits (Li et al., 2020).

The emergence of genome editing technology has accelerated the development of synthetic biology, but there are still some problems and room for improvement. At the same time, the application of this technology in the field of synthetic biology also needs further development.

### Methods of plant genetic transformation

A series of plant transformation systems have been developed: Agrobacterium-mediated method (Mayo et al., 2006; Zhang et al., 2006; Bahramnejad et al., 2019), particle bombardment (Ueki et al., 2009; Dong and Ronald, 2021), Electroporation (Furuhata et al., 2019) and Pollen-tube Pathway (Nagahara et al., 2021). However, these methods have some disadvantages, such as complex operation, long experiment period and few stable transformed species (Ramkumar et al., 2020). Due to the limitation of genotype, the use of gene editing is also limited for plants without a complete regeneration system. At present, most methods of plant genome modification involve tissue culture. The low transformation efficiency of plants is one of the bottlenecks in the development of phytoremediation.

Nanotechnology helps to efficiently and accurately deliver the required circuits to the chassis plants. Exogenous biomolecules can be internalized through the cell wall by nanomaterials without mechanical or external force assistance. Nanocarriers can effectively protect proteins, DNA, RNA and other biological molecules, and easily introduce target molecules into different tissues such as plant callus and endosperm. They have successfully mediated DNA transformation or delivered RNA to induce gene silencing in many plants (Pasupathy et al., 2008; Chang et al., 2013; Mitter et al., 2017; Kwak et al., 2019; Demirer et al., 2020; Lv et al., 2020; Schwartz et al., 2020; Zhang et al., 2021).

In the past ten years, through ectopic expression of developmental regulators (DR) such as BBM and Wus2, somatic embryo regeneration of some plants that cannot be transformed has been achieved to a certain extent (Boutilier et al., 2002; Passarinho et al., 2008; Che et al., 2022). Based on this, two methods based on Agrobacterium tumefaciens were established: Fast-TrACC (fast-treated Agrobacterium co-culture) and direct delivery (DD), which were used in dicotyledon plants to induce meristems by delivering Wus2 and BBM or other DRs involved in cytokinin synthesis to achieve genetic transformation. The operation of Fast-TrACC and DD is simple and time-consuming (Cody et al., 2022).

An extremely simple cut dip budding (CDB) system was created, which can easily and quickly obtain transgenic and genome editing plants without tissue culture under non sterile conditions. Based on root tillering, the system uses Agrobacterium rhizogenes to infect the cut root and stem junction to produce transformed roots, and then produces transformed buds through root transformation. This method realizes genetic transformation of multiple plant species. Moreover, this method has no genotype dependence.

### Challenges

#### Conduct basic research to reveal the mechanism controlling important processes

The accumulation of heavy metals in phytoextraction mainly includes the following processes: absorption of heavy metal ions by roots, transportation by apoplast and symplast, loading from root cells to xylem, long-distance transportation of xylem, unloading from xylem and transmembrane transportation of cells. It has been found that many genes are involved in different processes of the interaction between plants and heavy metals, including Heavy Metal ATPase (Huang et al., 2016), CDF (Cation Diffusion Facilitator) protein family (Yuan et al., 2012), phytochelatin synthase, metallothionein (Cobbett and Goldsbrough, 2002), etc. Plants activate various signaling pathways in response to heavy metal hazards (Mourato et al., 2015). The coating protein complex component Sec24C mediates the localization of the transporter ABCC1/2 to the vacuole through a Golgi-independent pathway, enabling it to play the role of vacuolar compartmentalization, and enhancing the tolerance of plants to heavy metal cadmium and arsenic stress (Wu et al., 2011). At present, the detoxification mechanisms of hyperaccumulators to heavy metals generally include the chelation of heavy metals by cytoplasmic substances, the repair of stress damage and the compartmentalization of vacuolar (Hall, 2002), but the detoxification mechanisms need to be further explored. Moreover, plants such as Viola baoshanensis (Shu et al., 2019), Sedum alfredii (Chen et al., 2020; Niu et al., 2021), *Leersia hexandra* Swartz (Liu et al., 2011), and *Pteris vittata (* Han et al., 2022) have the ability to repair heavy metal pollution, but few studies have been done on their specific regulatory mechanisms. The response mechanism of plants to heavy metals is complex, so we should deeply study the uptake, translocation, chelation and other mechanisms of super enriched plants to further promote the development of phytoremediation.

At present, the understanding of the process of plant synthesis of metal nanoparticles is still limited. Most studies are based on plant extracts, that is, the preparation of metal nanoparticles uses the method of plant tissue homogenate reacting with metal ions under certain environmental conditions. The results showed that the main substances related to plant synthesis of metal nanoparticles are organic acids, reducing sugars (Beattie and Haverkamp, 2011), proteins (Xie et al., 2007), amino acids (Shankar and Rhim, 2015) and peptides (Tan et al., 2010). However, the formation mechanism of metal nanoparticles cannot be fully revealed at the level of living plants.

#### Biosafety

With the rapid development of synthetic biology, it is becoming easier and easier to artificially transform or create life systems, and the biosafety of artificial life systems has become increasingly prominent. It is necessary to inhibit the escape of natural environment and malignant rapid growth of synthetic organisms, avoid gene invasion caused by horizontal transfer of artificial biological elements, and prevent artificial biosynthesis of toxic metabolites. Therefore, it is urgent to strengthen the research on the safety prevention and control of synthetic biology, so as to realize the knowability and controllability of the whole process of artificial life system, and provide security guarantee for the application of synthetic biology in the environmental field. In order to avoid the risk of integration of foreign fragments into the genome, Cas9 protein and gRNA were assembled into a ribonucleoprotein (RNP) *in vitro* for DNA-free genome editing, which has been successfully tested in many plants (Metje-Sprink et al., 2018).

## Conclusions and opinions

Phytoremediation is an effective method to control soil heavy metal pollution, but it is difficult to complete the remediation of complex pollution by a single plant at present. We propose that the remediation of soil heavy metal pollution should apply synthetic biology strategy, and the treatment process should use a comprehensive process of “microbial biosensor detection - phytoremediation - heavy metal recovery”. The heavy metal microbial biosensor should be integrated with microfluidic technology. The identification of hyperaccumulator species and other metal tolerant plants should continue, but local plant species should be used as much as possible to avoid the possible spread of invasive species. Moreover, chassis plants should be different from crops. The application of new experimental techniques will help to identify elements and redesign gene circuits.

The rapid development of synthetic biology has provided us with new technologies for creating modular and biological control systems. Accelerating the discovery of genetic elements and the artificial construction of gene circuits can improve the control effect of phytoremediation on soil heavy metal pollution.

## Author contributions

SB and DF designed the project and wrote the manuscript. XH revised the manuscript. All authors contributed to the article and approved the submitted version.
